# A Memoir on the Diseases Called by the People “Trembles,” and the “Sick-Stomach” or “Milk-Sickness;” as They Occurred in the Counties of Fayette, Madison, Clark, and Green in the State of Ohio

**Published:** 1841-03

**Authors:** Daniel Drake

**Affiliations:** The Medical Institute of Louisville


### WESTERN JOURNAL

OF

### MEDICINE AND SURGERY.

---

MARCH, 1841.

---

Art. I.—*A Memoir on the Diseases called by the people "Trembles," and the "Sick-Stomach" or "Milk-Sickness;" as they have occurred in the counties of Fayette, Madison, Clark, and Green in the State of Ohio.* Read before the Medical Convention of Kentucky, at Frankfort, January 12th, 1841. By Daniel Drake, M. D., of the Medical Institute of Louisville.

### INTRODUCTION.

It is generally known, that on the western side of the Alleghany mountains, from Georgia to the Lakes, a disorder, called *Trembles,* has for a long time prevailed among cattle and other domestic animals; and that wherever it has appeared, the people have been liable to a disease, which from its prominent symptom has received the name of *Sick-Stomach*, and from a hypothesis concerning its cause that of *Milk-Sickness.* These maladies, which may be regarded as endemics of the central portions of the Mississippi Valley, have not shown themselves equally in all parts of it—as large districts are known to be entirely exempt.

As far as we know, the first notice of these affections was published in Cincinnati in the year 1809,* and consisted in part, of the observations of Dr. Thomas Barbee, a highly intelligent and respectable physician of Bourbon county, in this state, long since deceased; who on a visit to the upper settlements on the Great Miami River in the state of Ohio, first became acquainted with it. Since that time, our medical and other journals have presented a great number of papers on the same subject, but notwithstanding the ability with which several of them have been drawn up, much obscurity and doubt still envelope it. The state of Kentucky, as liberal in her efforts to relieve the afflicted, as she has always proved herself gallant in the field, several years since offered a reward for the discovery of the remote cause or causes of these disorders, but no one has yet appeared successfully to claim the premium.

It cannot be doubted, that if *all* the infested places could be examined and compared, so as to ascertain the circumstances of soil, water and vegetable productions, in which they, at once, coincided with each other, and differed from the remainder of the country, the cause would be revealed. Such a comprehensive and minute survey, could best be made

* See Notices concerning Cincinnati.

by a single well qualified individual, who might for the requisite length of time, devote himself exclusively to it; but such an enterprise is not likely to be executed, and we shall, in all probability, have to wait for particular histories carefully drawn up and brought together for comparison. It was in the hope of making to the common stock, a contribution of this kind, that in the month of September last, we were induced to select for examination, a small district in the state of Ohio, which has long had the reputation of being greatly affected with these maladies. The time spent in it was from the 1st to the 15th of that month, during which we travelled on horseback and on foot, more than one hundred and fifty miles. We visited almost every important locality, conversed with a great number of physicians, who communicated their experience and observations with the utmost liberality; and made inquiry of a far greater number of respectable and intelligent farmers, living in situations favorable to this kind of investigation. In all cases, we sought by a rigid scrutiny to arrive at the truth, and often examined many different witnesses for the same fact. The answers were generally written down at the moment, or at the earliest practicable period afterwards; as were likewise all the observations made on the geology and botany of the region which we explored. No case of the disease, however, either in man or brute was met with. Information thus collected, although less authentic, and perhaps less accurate than that derived from personal observation, would seem to be not unworthy of the profession, and we have therefore embodied it in the following Memoir. We have in no instance incorporated with it published facts concerning these diseases, as they present themselves elsewhere, intending to give a monograph of them, as they show themselves, in the tract selected for examination. This we hope has been faithfully done, and with these preliminary remarks we shall proceed to lay them before the Convention.

### TOPOGRAPHY.

That part of the state of Ohio which is known as the Virginia Military District, is bounded south by the Ohio River, east by the Scioto, west by the Little Miami and north by the head waters of the Scioto, which lie so far to the west, as to be nearly in the same meridian with those of the Little Miami, which is the shorter river of the two. As they flow towards the south these small rivers diverge from each other, so as to enter the Ohio, nearly twice as far a part as they are at a middle point on the Scioto, in the latitude of 39 deg. and 45 min.—the mean latitude of the region to which our Memoir relates. The elevation of that region is about five hundred feet above the surface of Lake Erie, and something more than one thousand feet above the level of the ocean.

The southern tier of counties of the District are, to begin immediately below the mouth of the Scioto, Adams, Brown and Clermont, lying opposite to Lewis, Mason, Bracken, and Campbell counties in Kentucky. North of this tier lie a part of Pike and Ross, and the whole of Highland and Clinton. Then comes the entire county of Fayette, flanked with parts of Pickaway on the east, and Warren on the west. Immediately north of Fayette is Madison, with a portion of Franklin on the side next the Scioto, and of Green and Clark on that next the Miami. North of Madison lies Union. In the southern counties, the diseases under consideration occasionally show themselves in a few localities, but do not occur with frequency, till we enter the southern edge of Fayette, nearly in the latitude of Chillicothe; thence to the northern boundary of the District they are endemic, and, according to the reports of the people, have been of such frequent and fatal occurrence, as to render them subjects of deep public interest. Our exploration was limited to that part of the region which lies south of the national road, composed of the whole of Fayette, and parts of Madison, Clark, Green, Franklin, Pickaway and Ross. The surface of this portion of the District is so flat and level as to constitute it a table land. The streams which flow from it towards the Scioto, the Little Miami and Mad River, have channels so little inclined, that when low, the water almost stagnates, while from the same cause when, high they overflow their banks. After lagging for a time in these nearly horizontal beds, they approach the deeply cut channels of the Scioto and Little Miami, when their currents acquire greater velocity, and before they reach their destination, several of them present rapids, some even cataracts. In proportion as this occurs, the diseases we are investigating diminish in frequency, so that in the actual vallies of the two principal rivers, they are seldom met with. In fact they may be said to be limited to the table land. Confining our attention at present to it, we may note several varieties of surface. 1st. Prairies, which are the beds of shallow lakes or ponds, which have been gradually filled up, and the greater part or the whole of the water evaporated. 2d. A kind of maple swamps, or wet wood lands adjoining to the prairies, and nearly or quite on the same level with them. 3d. Barrens, also contiguous to the prairies, but generally a little elevated above them, with a poorer and dryer soil, presenting the remains of an oak forest, which had been thinned out by fire from the prairies. 4th. Heavily timbered oak lands, nearly on the same level with the last. 5th. Scattered through these oak lands, small wet places and shallow ponds, called by the people "slashes."* Of that kind of rolling, dry and fertile surface, which is presented by the central and north-eastern counties of Kentucky there is none.

### GEOLOGY.

That part of the Virginia Military District, which lies in the angle formed by the junction of the Scioto and Ohio rivers, presents the outcroppings of thick strata of conglomerate, topping the highest hills, and resting upon a fine grained sand stone; which itself reposes on a bed of soft, grey, friable argillaceous shale, which graduates into a bituminous slate. Immediately underneath this, appearing to the northwest of all the rest, is a compact, drab-greyish limestone, which stretches across the middle and northern part of the District. Over this formation, is every where spread a deep bed of diluvial or transported materials, consisting of blue and yellow clay, gravel and sand. Throughout the whole tract thus covered, there are but few springs; and of course the reliance of the people is upon wells, which vary in depth from five to forty feet. The water which they afford, though in general sufficiently transparent, is impregnated with the saline substances generally present in well water, rendering it hard; and it has, moreover, very often a sulphureous taste. In summer and autumn, when many of the smaller streams are dried up, the cattle and other domestic animals, which are kept in enclosed pastures, are watered from these wells,

* This is probably a corruption of slush, itself, a corruption of sludge. Being in universal use in the District, we shall adopt it into this Memoir.

while those which run at large drink the water of ponds and sluggish creeks.

### BOTANY.

1. The densely wooded TABLE-LANDS present, in great abundance, several species of oak—(white, black, and red in the language of the people)—such as *quercus alba, tinctoria, falcata* and others. Intermingled with these are two or three hickories (*carya;*) the tulip or yellow poplar tree (*lir: tulipifera;*) dog-wood (*cornus florida;*) black ash (*fraxinus sambucifolia;*) sassafras and spicewood (*laurus;*) plums and cherries (*prunus;*) hazle (*corylus;*) black-haw (*viburnum;*) and other trees, shrubs and vines, which in all parts of the West are found in natural association with these.

As far as we had time and skill to examine the herbaceous flora of these tables, as it presented itself in autumn, we found it in harmony with the arborescent. Not a single plant fell under our notice, which we had not been accustomed to see in the corresponding oak lands of Ohio and Kentucky; an observation that conforms entirely, with the more accurate and careful inquiries of Dr. Riddell, Dr. Warder, and Mr. Sullivant; the last of whom expressly declares, that he has not been able to find, on those plateaus, one species of plant which is peculiar to them.

2. The SLASHES, or small wet places, interspersed through the plateaus, present a striking modification of the surrounding flora; as most of the plants found on the latter, are replaced by those which delight in a deep, black, wet soil. The characteristic trees are the white elm (*ulmus Americana;*) black walnut (*juglans nigra;*) burr oak (*q. macrocarpa;*) honey locust (*gleditsia triacanthos;*) water maple (*acer rubrum;*) and black ash (*fraxinus.*) Of the climbing vines, the principal are several species of *smilax;* the *celastrus scandens,* an *occasional* grape (*vitis;*) ivy (*ampelopsis quinquefolia;*) creeper (*bignonia capreolata;*) above all, the poison vine (*rhus radicans et toxicodendron,*) which although common enough in the adjoining oak lands, is far more frequent and luxuriant, in and around the SLASHES, than we have ever seen it elsewhere. It *seems* to select the white elm, and like a parasite clings to almost every individual of that species. Of the shrubs found in these spots, we may mention the spice-wood (*laurus,*) in a few, the poison or swamp dog-wood (*rhus venenata, olim vernix,*) and the button head (*cephalanthus occidentalis.*)

The herbaceous botany of these little localities, differs from that of the surrounding, drier table-lands chiefly in this, that while species which flourish best in a wet rich soil, show comparatively but a few individuals, on the general surface of the plateaus, they here present a great number—those which are natural to thin and dry surfaces disappearing. Thus, for example, the *lobelias—syphilica and cardinalis,* are in these places extremely common, but the *inflata* rare; the *ranunculi* become abundant; and the *eupatorium ageratoides,* which grows in profusion over the adjoining oak lands, is replaced by the *e. perfoliatum.* Of herbaceous plants *peculiar* to these spots we did not find a single species.

3. The BARRENS present much less timber and fewer species of every kind, as they have been destroyed by fire. Oak and hickory are the predominant forest trees, and hazle bushes the chief shrubbery. The *rhus* and the other climbing vines, with the exception of the *celastrus scandens,* are rare. Different grasses have taken the places of many other herbaceous plants, the latter having been destroyed by ancient conflagrations. In short, we have here the surviving vegetations of the plateaus; but we need not dwell on the botany of these tracts, as they are known *not* to give rise to the diseases we are investigating.

4 and 5. The prairies and adjoining flats, are universally believed to be equally exempt from the cause of those maladies and we shall not, therefore, dwell on their botany.

With this sketch of the topography, geology and botany of the district to which we have invited attention, let us proceed to speak of the diseases which are the immediate subjects of our inquiry; in doing which, not a single fact not observed by ourselves or others, *within the District*, will be introduced. We shall begin with the epizootic malady, known only by the name of

### THE TREMBLES.

The following animals are enumerated as subject to this malady: 1, the cow; 2, the horse; 3, the sheep; 4, the hog; 5, the dog; to which may be added as doubtful, 6, the goat; 7, the mule; 8, the buzzard. Each of these names is used for a species, and not restricted, either to sex, or any group of individuals. The characteristic symptoms are so much alike in all, that an account of them in one of the species will serve, with a few variations and additions, for the whole. When they are stated, the members of the Convention will judge for themselves, whether, in its symptomatology, this affection presents any thing peculiar. We select the first, as the most liable to the disease, or at least in which it has been oftenest and best observed.

### *The Cow.*

In the earliest stages of this malady, in the cow, it may not display its existence, if the attack be not violent and the animal left to itself; for in the beginning, as in all stages of the disorder, the appetite seems to be unimpaired, and the thirst not increased. Even this early stage not less than the more advanced, appears, however, to be attended with constipation of the bowels. The animal at length begins to mope and droop, to walk slower than its fellows, and to falter in its gait. If under these circumstances, it should be driven, and attempt to run, the debility and stiffness of its muscles are immediately apparent. It fails rapidly, trembles, pants, and sometimes seems blind, as it runs against obstacles, but this may arise from vertigo; at length it falls down, lies on its side quivering, and is not, perhaps, able to rise for several hours, sometimes never. Now and then, the quivering amounts to a slight convulsion. When the disease is not violent, the animal after a longer or shorter period, is again on its feet; but its capacity for muscular effort is greatly impaired, and, if hurried in the slightest degree, it is siezed with trembling and stiffness, and may even fall again. Of the state of the circulation, when it lies seriously ill, but little is known, as the pulse has not been inspected. One observer perceived that the nose of a heifer was hot, but others have found that part and the skin generally cool. Perhaps their observations were made in different stages of the disease. While lying unable to walk, the animal will still eat freely, and also take drink, but does not seem to have excessive thirst. Its costiveness continues to the last when the malady goes on to a fatal termination. Of the symptoms which precede dissolution, we could not obtain a satisfactory account. Our witnesses generally declared, however, that the abdomen does not swell in any stage of the disease. When it assumes a chronic form, the animal is liable, for weeks and even months, to muscular infirmity under exercise, looks gaunt and thin, its hair assumes a dead appearance, and sometimes falls off in considerable quantities, especially from the neck.

Males and females are said to be equally liable, except such of the latter as give milk. To their exemption, every individual whom we interrogated bore testimony. Sucking calves are exceedingly liable to the malady. If driven rapidly, they fall and tremble; but the paroxysm generally comes on when they are at the teat. They suck for a minute or more, then stagger off and fall, struggle, are sometimes slightly convulsed, and a quantity of milk is regurgitated from the pharynx or stomach. After reposing for a short time, they rise and resume the teat, but perhaps fall again. The people ascribe these paroxysms to the impress of the milk upon the stomach of the little animal; but a more probable explanation is, that they are excited by the muscular efforts made in sucking, after ten or twelve hours separation from the cow. Calves often die of this malady, and their dams are said, then, frequently to become affected with the disease. The milk has no peculiar odor, and is readily formed into butter and cheese.

The *pathological anatomy* of this disorder has not yet been *studied*. The medical gentlemen where it prevails have certainly been remiss on this point, as they have had many opportunities of making *post mortem* examinations. Dr. Toland, a respectable physician of London, in Madison county, has, however, inspected the abdominal viscera of several cattle dead from this malady. The contents of the maniplies or third stomach were dry and hard, its mucous membrane was dry and seemed to adhere to the alimentary mass, with which it would come away, though it did not appear to be mortified. The mucous membrane of the small intestines was, in some places dry, in others greatly injected with blood; their contents were hardened; they were not distended with gas. The gall bladder was filled with black, pitchy bile. In some cases, the kidneys were nearly black, and appeared to be gangrenous. On opening the carcases (before putrefaction had commenced) an offensive odor was emitted not unlike that attendant on a mercurial salivation; an observation which was confirmed by that of Lewis Skilling, who lives near South Charleston, in Clark county. He lost twenty-one cattle about one time, and found, on skinning them, that every one emitted an offensive smell of a peculiar kind, before the cavities were opened. They made candles of some of the tallow, but in burning, its smell was so offensive, that they were obliged to throw them away. Daniel Horney, near Jeffersonville, Fayette county, has found the contents of the alimentary canal dry and hard. Mr. Keller, near Martinsburg, in the same county, made one examination and found the contents dry as saw dust. Jonathan Pierce, near South Charleston, opened some of his cattle dead from this disease, and found the contents of the maniplies dry; and those of about a foot of the bowels likewise, the mucous membrane of the latter being red.

### *The Horse.*

The symptoms in the horse are substantially the same as in the cow. It often happens, that he is not known to be in the forming stage, or even predisposed to the malady, when, on pushing him for a short distance under the saddle, or riding for half a day on a journey, or driving him in a team, he begins to sweat profusely, lags, trembles, breathes laboriously, and if urged on, falls down and lies trembling. In this situation, he sometimes suddenly dies, at other times revives, and if not required to travel home may recover. He is costive like the cow. The mare while nursing, is exempt, and her colt liable like the calf. It sometimes, while sucking, ejects the milk from its mouth and nostrils. Horses, in proportion to their number, and their being kept under the same circumstances as horned cattle, appear to be equally liable.

### *Sheep.*

These animals so often die, in the District, of other diseases, that some confusion exists in the reports on their symptoms. Comparing all that were made to us, we are prepared to say, that the symptoms displayed by them in the muscular system, and in the bowels, are the same as those already described. Lambs are subject to the disease, but their mothers generally escape till after they are weaned.

### *Hogs.*

Hogs, considering their number, are less frequently affected than cattle, horses and sheep. They have the same muscular enervation with other animals, lose their flesh, become costive, and often utter low squeaks or grunts, indicative of distress. It has some times been observed, that the breath of pigs affected with the malady was altered and offensive; an observation which only those who are accustomed to make house pets of these animals, could be qualified to make.

### *The Dog.*

When this animal is attacked, he loses his watchfulness, and lies about utterly indisposed to action. When urged to move, he is found to be stiff, and after faltering and vacillating, falls or lies down. He eats, but loses flesh, and is costive. In this condition he may remain a while and recover; but if stimulated to a vigorous muscular effort, he sometimes suddenly expires. Judge Jameson, near Washington, Fayette county, had a dog laboring under a mild attack, who started after a deer, that was crossing the farm. He ran with considerable speed for about two hundred yards, when he suddenly stopped, uttered a yell and fell down, and soon afterwards expired. Henry Massie, Esq., knew of a case, in which a dog affected with the disease was drawn into a fight with another, and in the midst of it expired. William Janes, of Jeffersonville, in the same county, has several times seen dogs "drop down dead," when running after animals. Dogs are sometimes observed to vomit. Their temper is not irascible. We did not learn whether, the bitch which gives suck is exempt—the puppies are not.

### *The Goat.*

We heard of but one case of the disease in this animal, which, however, is rare in the District and keeps about the houses and yards.

### *The Mule.*

As but few mules are grown in the District, but little is known as to their liability. We could learn of but one death of this tribe. The symptoms were said to be the same that have been enumerated.

### *The Buzzard.*

Three persons have seen buzzards, under circumstances which cannot be made intelligible in this part of our Memoir, that suggested to the observers, that these birds were affected with the disease. Their symptoms were muscular.

### TREATMENT OF THE TREMBLES.

We met with no medical gentleman, who had subjected animals laboring under this disease to a systematic, or even varied empirical treatment. All the people of the District have one and the same indication to fulfil—that of opening the bowels. When this can be effected, the animal, they say, scarcely ever dies—when it cannot, death occurs. For the fulfilment of this indication, Epsom salt has been administered in very large quantities, even to pounds, but without effect. Drenches of lard and various mixtures, have also been given, with no satisfactory result. Judge Harold, near South Charleston, has exhibited calomel followed by lard—no essential benefit. Dr. Toland has administered the oil of turpentine, in doses of eight, twelve and sixteen ounces, without advantage. An opinion is prevalent, that drenching animals injures them by causing them to struggle. On the whole, we found among the people of the District, a total want of confidence, in all kinds of cathartic *medicines;* and an exclusive reliance on Indian corn. Some preferred old corn, some new, and others that which had been frost bitten. This is fed to all those species of animals that are accustomed to eat it, and is said never to be refused. The more the animal will eat, the greater is the hope of the owner. It is said to produce *purging*, when every other means have failed, and then, it is affirmed, recovery is almost certain. On these points we found but one opinion in the District. Several of its physicians, after trying other things, had, with the people, settled down on this.

We found blood letting not in favor. Dr. Toland supposes it has, generally, been employed at too late a period. Many non-professional persons, spoke of having resorted to it without advantage, and some thought it had done harm.

Throughout the disease, rest is considered a *sine qua non*, to the favorable effect of any measure; and of itself, in mild cases, sufficient; that is, if they be not aggravated by exercise, the disease will wear itself out, or spontaneously subside.

### OF THE SICK-STOMACH OR MILK-SICKNESS.

Of the popular opinion, that this malady has in its cause a connexion with the Trembles, we shall speak presently. It attacks men, women and children, in an equal degree; young children, however, are said to be less liable.

When the individual is about to be taken down, he feels weary, trembles more or less under exertion, and often experiences pain, numbness and slight cramps in the calves and other muscles of his legs. At the same time, he becomes costive; and, under fatigue, is likely to experience a slight degree of nausea. His appetite is not generally impaired, but he has a feeling of depression and burning, at the pit of the stomach. He is irresolute, and as much indisposed to mental as to bodily effort. He may continue in this situation for a while and recover spontaneously, or by a single cathartic; but more commonly under the influence of an exciting cause, severe symptoms supervene. A full meal, or a great quantity of indigestible food is such an one; but another, far more common and efficient, is violent or protracted muscular exertion. This is, indeed, the only exciting cause, which all the people and physicians of the District, concur in admitting. The patient being subjected to this, full vomiting supervenes, with much epigastric distress. He throws up the contents of his stomach; and continuing, at short intervals, to vomit or retch, brings up small quantities of acid and mucus, but very seldom much bile. In attempting to sit up, his sickness increases, and his muscles generally become affected with a twitching and tumultuary motion. In lying he is restless, and tosses himself from side to side, in great anxiety. His thirst is generally unquenchable. In the midst of these symptoms, his bowels remain torpid, without pain, and do not swell, but seem rather reduced in volume, with a retraction of the umbilicus towards the spine; so that the pulsations of the aorta can be distinctly perceived. Dr. Thos. McGarraugh, now of Frankfort, Ross county, but for many years an observing practitioner, of Washington, Fayette county, states, that the tongue is sometimes natural, but at others pale and covered with a film of mucus, which is yellow in the middle; in the advanced stages of certain violent cases, it looks like a mass of raw flesh. The heart manifests a convulsive action, and the aorta pulsates with unwonted violence. Notwithstanding all this, the pulse is sometimes almost natural, and although in certain cases increased in tension, is not apt to be very frequent. Now and then its diameter becomes very small. These are the remarks of Dr. Toland, which in the main, correspond with those of other observers. There is no cough; but the patient sighs, and his breath has a singular, offensive, somewhat mercurial odor; so distinct from that in autumnal fever or any other known disease, as to be regarded quite characteristic of this. The skin is generally cool, in the latter stages cold; Dr. McGarraugh has, however, in some cases observed increased heat on the thorax. The surface generally, is always dry.

Many cases are cured, when the symptoms here enumerated are present; but others go on to a fatal termination. In such, the irritability of the stomach is often so great, that the least motion in bed, or a moment's conversation, will bring on retching, or vomiting; when, in several instances, the patient has thrown up a dark colored matter, like that ejected in fatal cases of yellow fever;—death does not however, so constantly follow this discharge, as it does, the coffee ground evacuation, in that disease. The constipation continues, and the retraction of the umbilicus rather increases than diminishes. There is no peritoneal soreness on pressure; even the epigastrium does not appear to be particularly tender, but pressure upon it, is apt to excite vomiting. The sighing becomes deeper, and a hiccough supervenes, which is not always followed by death. The pulse gradually sinks in fulness and force, but the struggling movement of the heart, and the convulsive or throbbing action of the aorta are rather augmented than abated. The visage of the patient, seldom red in the earlier stages, now becomes deathly pale and shrunk up. He tosses less, becomes listless, indifferent to what is passing round him, and often lies, between his fits of retching, in a mild coma. Very seldom is there any delirium; he answers well, and does not often mutter when left to himself; there is, however, a peculiar feebleness of attention, inasmuch as many, who have recovered from this advanced stage of the disease, and were supposed, at the time, to be cognizant of all that passed around them, were found afterwards not to remember much of what had transpired; not even the death of relatives, who died from the same disease in the same rooms with themselves. We need not follow these symptoms further, as they bring us to that stage when, from the approach of death, the phenomena must be nearly the same, as in the close of all other maladies. The length of time through which the disease runs, to its termination in recovery or death, varies exceedingly in different cases. In some it comes to a favorable termination within twenty-four hours after the vomiting sets in;—again, that with the other symptoms, continues violent for a week or more, and then ceases. Death, also, occurs at every period from the second or third day, up to the end of the second week. Dr. M. Winans, of Jamestown, Green county, who has had an ample experience in the complaint, thinks that the convalescence from it, is generally rapid; however this may be in many cases, it is an established fact in the District, that relapses, under exercise, are common, and that the patient often remains tremulous, feeble, and stiff in his muscular system, for months; feeling irresolute and destitute of enterprise, and experiencing a slight degree of nausea under any great exertion. We were more than once told by healthy looking individuals, who had experienced the disease some years previously, that they had never since been the "same men they were before." In a few instances, the complaint has recurred for two or three years, in the season in which it began, without any known reapplication of the remote cause.

As yet we have considered the sick-stomach in its most simple aspect. We must now say something of its complication.

Several medical gentlemen of the District, have met with cases, in which some variety of cerebral inflammation supervened, as was indicated, by a flushed face, red eyes and delirium.

Dr. McGarraugh has seen it combined with ague and fever in two patients. Each disease exhibited its characteristic symptoms—the former continuing throughout the twenty-four hours, the latter intermitting. They both died.

Dr. Toland has met with it in connexion with remitting fever; the disease commencing as the Sick-Stomach, and ending as bilious fever, but the number of these cases has not been great.

The same gentleman with Dr. Robert Houston, of Clark county, and some other physicians of the District, have observed within the last two or three years, that it has been frequently complicated with a variety of mild typhus or typhoid fever; not a little of which has prevailed in localities where the Sick-Stomach does not occur. This combination has modified its symptoms, and increased the difficulty of treating it successfully.

### TREATMENT.

The catalogue of remedies employed in this disease, when uninvolved with any other, is not very extensive. We shall enumerate the principal.

1. *Bloodletting.* Although from its general priority, to other means in the cure of many acute diseases, we place this first, it is, by no means, that on which the chief reliance is placed by the physicians and people of the District. Indeed, not a few of them almost reject it, as they condemn it in the treatment of the Trembles. It is no part of our plan to speculate on this condemnation, as we wish to limit ourselves to facts; and, especially, as some of the most intelligent practitioners of the District frequently employ it.

Thus, Dr. McGarraugh has bled about one-third of his patients. The blood was sometimes buffy, in general not. When the pulse was full and hard, and not very frequent, venesection was most advantageous; when small, it seldom increased in fulness and force, after bleeding. He has not often bled more than once, and then took, in general, about a pint. He found his patients very often inclined to syncope. It was chiefly when some affection of the brain existed, that he took blood.

Dr. Toland has bled less frequently. He has observed the blood, in some cases, to be sizy. His resort to the lancet was almost limited to the cases in which he observed the brain to be affected. Dr. Houston has bled in a few cases only, and does not think highly of the remedy; the same is true of Dr. E. Martin, of Bloomingsburg, Fayette county. Dr. E. Crosby of the same place has never bled; Dr. Winans not very often.

Dr. James M. P. Baskerville, late of Madison county, thinks blood letting of doubtful utility. Has found the blood thick, dark, disposed to early coagulation and without size. The pulse has sunk after bleeding.

These gentlemen may be considered as presenting the estimate of blood letting, made by the physicians of the District generally. When the people treat the disease, they scarcely ever resort to the lancet. Cupping and leeching of the epigastrium seem not to have been employed.

2. *Cathartics.* These are the remedies which are universally employed. We need not state the individual experience of the physicians. Both they and the people, make the indication of opening the bowels—*the* indication of *cure;* and affirm, that when *it* is accomplished, the patient rapidly recovers. In the forming stages of the disease, this is, in general, easily brought about, and violent symptoms averted. After the vomiting has commenced, the difficulty is greatly increased; as the medicines are ejected from the stomach. It seems to be the experience of the other physicians of the District, as well as of Dr. Baskerville, that bleeding does not promote the operation of cathartics, as in many other acute diseases. Calomel is the medicine, on which the chief reliance is placed. It lies best on the stomach, and is generally given in large and repeated doses. When the patient recovers, a salivation occasionally supervenes. In connexion with this medicine, sulphate of magnesia, senna, castor oil and oil of turpentine have been used, and are thought the best. Croton oil has succeeded in a case or two in Dr. Dawson's hands, but in general disappointed the expectation under which it had been administered. Enemata have been almost constantly used, and often done good, though in many cases they have been returned without effecting the object. The length of time through which the costiveness continues, is sometimes surprisingly great. A respectable merchant, in Washington, Fayette county, who had all the symptoms of the disease, took many large doses of calomel, daily, without the least effect till the fifth day. He then resorted to Seidlitz powders, when the vomiting ceased, and a purging and salivation came on simultaneously. In many other instances, the constipation has continued still longer, and yet the patient recovered. Thus Dr. Toland attended a youth, 17 years old, whose costiveness continued for fourteen days, and still he recovered. When cathartics at last operate the discharges are not scybalous but dark and liquid.

3. *Opium.* Much reliance is placed on this medicine. It is often given before any thing else, to allay the irritability of the stomach and prepare it for the retention of calomel. It has been combined, advantageously, with that medicine. It has not appeared to increase the constipation.

4. *Counter-irritants.* Blisters, sinapisms and oil of turpentine, have all been applied to the epigastrium; and the weight of testimony is in their favor. Blisters to the ankles have likewise been employed with some advantage.

5. *Cold affusions.* When the head is much affected, either from the disease, or from the liberal use of opium, cold water applied to it, has been of much service. Dr. Toland has, also, subjected his patients to the general cold dash, with advantage. In the case of the patient already mentioned, whose costiveness contined for a fortnight, purging followed on the cold affusion. The Doctor is in the habit of applying external heat, immediately after the cold water, and is thus sometimes enabled to induce perspiration.

6. *Antacids.* These have been used, but not with decided benefit.

7. *Alcoholic tinctures* and other *diffusible stimuli*, seem to have been employed but seldom. In some cases they have been of service.

8. *Drinks.* Dr. McGarraugh has sometimes found demulcent drinks of service. A coffee made of scorched oats, an infusion of wheat, and weak chicken broth have all proved useful.

9. *Treatment of the Convalescent.* The action of the bowels must be kept up, and hearty meals avoided: but above all the patient must refrain from violent exercise. Dr. Toland had a patient, a girl, so far restored, that she ran down a chicken for the purpose of making broth. She suddenly relapsed, and died in twenty-four hours. Dr. McGarraugh has found tonics of service, when the recovery was slow, and thinks chalybeates better than bitters.

Without having exhausted our collection of facts we shall here take leave of the symptoms and treatment of this malady. The members of the Convention will judge for themselves how far it has claims to be regarded as a peculiar disease, and frame their own speculations on its pathology. Our object is to present facts.

### CAUSE OF TREMBLES IN HERBIVOROUS ANIMALS.

In speaking of the topographical aspects of the District, we recognized six varieties,—first, prairies—second, flats adjoining these—third, barrens—fourth, hills near the Scioto and Little Miami rivers, and along their longer tributaries—fifth, table-lands covered with heavy oak timber—sixth, slashes or superficial ponds, marshes and wet places, of small individual extent, interspersed through the table-lands, from which they are readily distinguished by a growth of elm, black walnut and burr oak. Now it is the belief, we may say of all the people of the District, that the disease is never generated on the four former, for, it is asserted, as long as the domestic, herbivorous animals are confined to them, they never experience it. The disease originates then, according to this statement, only in the oak table-lands, abounding in elm and walnut slashes. If this be established, one step towards the discovery of the remote cause is made; but is this assertion of the people correct? That it is, we shall endeavor to show, by the citation of testimony which goes to prove, not only the exemption of the former tracts, but the actual influence of the latter in the production of the endemic.

In our exploration of the District, whenever we traversed the prairies and adjoining flats, or barrens, or hill lands near the rivers, we could not meet with a single person, who had known the disease to occur, in those situations; on the other hand, whenever we came into or near the oak plateaus, every body, not only admitted its existence there, but equally admitted, that as far as he knew or had heard, the other varieties of surface did not produce it. Thus giving testimony against the salubrity of his own lands, and thereby affording satisfactory evidence of his sincerity. On the question, whether the cause is connected with the oak lands themselves, or the elm slashes which they embosom, we found some diversity of opinion, but a large majority fixed the mischief upon the latter, still further narrowing down the field of inquiry, if they should be correct. We shall now proceed to the citation of particular observations and facts, which have a bearing on the whole of these conclusions, beginning with those furnished us by the medical gentlemen of the District.

Dr. Winans, of Jamestown, who has continued his observations for eighteen years in the eastern portions of Green and the western parts of Fayette county, declares, that the disease never prevails except in and near the heavily timbered oak lands; that it is not generated in the prairies, flats, barrens or hills, and that it ceases when the forest is cut down.

Dr. Dawson of the same place, a native of the county, from his own observations has come to the same conclusion.

These gentlemen furnished us with several pertinent facts, two of which we shall give. To the east of Jamestown, there lies an oak plateau, several miles in width, running nearly north and south, and seperating the waters which fall into the Little Miami on the west, from those which flow to the Scioto on the east. This plateau, as those gentlemen testify, and as we ascertained by personal inspection, abounds in elm slashes. In the early settlement of the country, from its elevation of twenty or thirty feet above the valleys on either side, it was supposed to be more salubrious, and many emigrants selected it on that account. It was soon found, however, that their cattle and other domestic animals contracted the Trembles; while those of the settlers in the adjoining valleys remained exempt, except when they wandered into the dense oak forests of the plateau. We shall give two facts in support of this statement.

Dr. Winans, who lives in the valley to the west, some years since, having a farm on the plateau, sent to it a drove of hogs which were to be fed near its enclosures, and suffered to run at large. They were all well, and none of them had ever had the Trembles. In a fortnight many of them wandered back to the village, affected with the disease.

Many years ago a herd of cattle, pastured in the barrens east of this plateau, where the disease has never occurred, escaped from their enclosure, and strayed upon it, where they were allowed to feed for eight or ten days, when they were taken home, and a large proportion of them soon afterwards died of the Trembles.

Dr. Houston, after a residence of twenty years, at South Charleston, assured us, that the disease, so far as herbivorous animals are concerned, is, in that vicinity, limited to flat oak lands, which abounds in wet places, producing elm and black walnut. Prairies and barrens are numerous within the circle of his practice, and always exempt.

Dr. Henton, of Washington, Fayette county, who has for many years had ample opportunities of observation, assured us, that the disease prevails in, and is limited to, regions abounding in rich, marshy spots, where elm, maple and burr oak show themselves. South-west and west of Washington, much of this surface exists, and the disease has been and still is common and fatal; while south-east and east, to the Scioto, as the land becomes broken it is almost unknown. He has never seen it occur among animals confined to the prairies and barrens.

Dr. McGarraugh, through a still longer residence at the same place, has observed precisely the same thing. The barrens and prairies are exempt, the plateaus of heavily timbered land abounding in elm and walnut slashes, are infested.

Dr. Baskerville, formerly of Madison county, gave us the same result from his observations.

Dr. Bradbury, at present of Ross, but formerly of Clark county, who saw much of the disease, when residing there for five years, declared that the prairies and barrens are free from it, and that it is generated in the flat, wet, heavy timbered lands, abounding in spots covered with white elm and burr oak. Since his removal forty miles to the south-east, into the hilly or rolling land of the Scioto, he has never met with it.

Dr. Martin, of Bloomingsburg, who resided for some time several miles to the east, where the land is more broken, saw but little of the disease; but is familiar with it now, and knows that it is limited in its origin, to the oak forests with elm and maple slashes.

Dr. Crosby, of the same place, after, however, a shorter residence, has come to precisely the same conclusion.

Dr. Dunlap, of Greenfield, resides nearly out of the infected region, as the lands to the south of him are more rolling and dry. All the cases he has known, were to the north, where the varieties of surface so often enumerated exist.

Dr. Wright, of Leesburg, west of Greenfield, has made similar observations.

Dr. Toland, after a practice of twenty years in Madison county, a few miles south of the national road, declares that it is never generated elsewhere than in the heavily timbered flat lands. He travelled with us into a tract, distinguished, as he and the inhabitants assured us, for the production of the disease, and we found the elm, walnut and burr oak slashes, more numerous than we had seen them before. This was west and north-west of London, where he resides—south-east of the town, prairies and barrens abound, and there the disease is almost unknown.

We have thus given the testimony of twelve medical gentlemen, who have for several years been the principal physicians of the District, and, throughout the whole period, had their attention strongly directed to this subject. Scattered over a region, more than forty miles in diameter, they have had but little intercourse, and many of them, as we found, had never seen each other. They have not, therefore, adopted the conclusions of any one, but each has seen for himself, that which the circle of his own observations presented.

Such a mass of evidence might seem sufficient; but as we regard the establishment of this point of much importance, we shall introduce some additional witnesses from among the people.

On the day before leaving Chillicothe, we received from Cadwalleder Wallace, Esq., of that place, a gentleman whose business has long made him familiar with the condition of the District, and a close observer, a communication, in which he expressly declares, that the prairies, barrens and cultivated lands never breed the disease, but that it is generated in the slashy, timbered lands.

Judge Jameson, who has resided for the last twenty-five years, about two miles south-west of Washington, in Fayette county, lived for many years before, in Ross county, twenty-four miles to the south-east of his present residence. He was then in rolling or broken land, and never witnessed a case of the disease. He is now in the midst of elm and maple slashes, and his stock, when permitted to run at large, have suffered from the time he arrived, down to the present. More than once he lost all his horses, before he had pastures enclosed. Of his cattle many have likewise died; but none of them perished in the prairies, barrens or cultivated fields. Among particular facts received from him, we give the following, which was substantiated, independently of his own unquestionable testimony, by that of several other persons. About twenty years ago, being a grazier, he purchased about one hundred head of cattle from his neighbors, and seperated the younger and more infirm, to the number of about thirty, from the rest, and turned them into a new meadow, formed out of elm and maple slashes. Most of the trees had been felled, and the ground harrowed, but not plowed. The sown grass had taken root two years before. The remaining seventy, he placed on the opposite of a narrow lane, in a wood-pasture, the surface of which was precisely of the kind which the meadow *had* been. The whole were salted at the same time, and watered from the same well. Nothing was fed to any of them. On a certain day, in salting them, they all seemed perfectly well in both fields; but the very next day, between thirty and forty, or about one-half of those running in the woodland pasture, were found to have the Trembles. He immediately turned all that remained well, into the meadow, where four or five of them afterwards sickened with the same disease. Those which were ill, he turned into a corn field, and fed them with unripe indian corn. Nineteen of them died, while not an individual, originally placed in the meadow had the disease.

Jonathan Pierce, who has an extensive grazing farm for cattle and sheep, in the south-eastern part of Clark county, gave us the following fact. Two years ago last fall, thirty of his cattle broke from a *tame* pasture, where the Trembles had never been bred, and browsed for a few days, in a piece of thickly timbered oak land, abounding as we ourselves saw, in elm slashes. Nine were seized with the disease, and four died. He has had ample opportunities of knowing, that cattle may run with impunity in the barrens and prairies.

Judge Harold, a neighbor of Mr. Pierce, communicated to us the following fact. Twelve or fourteen years ago, he enclosed forty-five acres of wild land, about one-third of which was prairie, another part barren, the remainder closely timbered land, with a pond and several elm slashes, precisely like those on which Mr. Pierce's cattle were destroyed. He pastured his stock in this field for six years, without the occurrence of the disease, though they ate down the herbage in the woodland as well as in the other parts. In the seventh year, he determined to mow the prairie, and of course withdrew his stock. The natural herbage of the woodlands now sprang up, in rank luxuriance. Immediately after mowing the prairie, when it could afford no pasture, he turned in ten head of horses, twelve of cattle, and sixty of sheep. Within a fortnight, several of the horses sickened with the disease, and two or three died; of the cattle, about the same number perished, with the same symptoms; and of the sheep, twelve or fourteen were lost. The next spring, he turned in a large flock of sheep which kept the grass and herbage eaten down, from the time they began to grow, and he, also, deadened the timber, in and around the flats, which he plowed up. A few sheep died within the next two or three years, but since that time, it has been as safe as any other pasture on his farm.

When travelling from London to Springfield, a few miles south of the national road, over the elevated and heavily timbered plateau, which seperates the waters of several branches of the Scioto, Mad river and Little Miami, we conversed with a number of intelligent farmers, among whom we may mention James Porter, Robert Turner and Enoch King, all of whom testified to the extraordinary prevalence of Trembles in that quarter; and we can state from personal observation, that we met with no spot in the District, which in an equal area presented so great a number of elm slashes.

We consider it unnecessary to quote, specifically, the testimony of any other observers, as the whole would tend to the same point.

We feel warranted, then, in deducing and resting upon the following conclusions:

1. That in *this District*, the Trembles in cattle, horses, sheep and hogs, are produced by their frequenting the densely timbered table-land, which from its flatness abounds in wet places and ponds, indicated by the presence of lofty white elms, black walnuts, maples, burr oaks, and other trees, which delight in a rich and moist soil.

2. That when the same animals frequent prairies, barrens, and the hill-lands, near the larger streams, although they may be heavily timbered, they do not contract it; and, consequently, its cause does not exist there, or at least is not efficient.

3. That clearing and cultivation, even girdling the trees, harrowing the ground, and sowing it with grass seed, destroys or renders inactive the cause, whatever it may be.

To these conclusions we add the following, as supported by a multitude of witnesses:

1. That the disease in the same localities, is not equally prevalent every year.

2. That it occasionally occurs in May and June, but its usual time of prevalence, is August, September, October and November.

3. That in proportion to the number of domestic animals now in the District, it is much less prevalent than formerly; and that there has been a regular decrease of it, as the forest was transformed into arable land, and stock were kept within enclosed meadows.

### OF THE CAUSE OF SICK-STOMACH OR MILK-SICKNESS, AND THE CONNEXION OF THAT DISEASE WITH THE TREMBLES.

But one *opinion* prevails throughout the District, as to the cause of Sick-Stomach. Both the physicians and people regard it, as an effect of eating the milk or flesh of animals, whose systems have been impressed with, or have received into them, the remote cause of Trembles. An opinion so general and firmly established, in the minds of those who cherish and express it, is undoubtedly entitled to much respect; and, if it did not contravene what are regarded as established physiological principles, might even be adopted without inquiry into the facts on which it rests. Such an adoption is not, however, admissible here; and we must proceed to ascertain the data from which it has been deduced, that we may judge of their sufficiency.

In doing this, the first that meets us, is the perfect co-existence of the two maladies in time and place. Taken as a general proportion, we are satisfied, from a vast variety of testimony, that this is true; while cases of each malady, occasionally show themselves, when the other is absent, no satisfactory explanation of which can be given in this stage of our enquiry. But the co-existence of two phenomena, does not prove their origin from the same cause, nor of either one from the other. Nevertheless, that this fact deserves consideration, is apparent, from the reflection, that if such a co-existence were wanting, the opinion which prevails would never have been formed, and could not, indeed be entertained. We shall take this, then, as the starting point. The physicians and the people generally of the District, affirm, that in almost every case, which has presented itself, the patient had taken the milk, or its preparations, or the flesh of cattle which had frequented the spots which are known to produce Trembles. Several of the most intelligent of the medical gentlemen assured us, that they had been utterly incredulous on this point, but had been compelled, under continued observation to concur in the popular belief. It is regarded in the District, as an equally ascertained fact, that when milch cows are kept on meadows, those who use their milk, and do not eat the flesh of such as run in the dense woods, never have the disease, however much it may prevail at the time, among others, who have suffered their cattle to go at large. That milch cows, which frequent spots where Trembles are generated, are uniformly, or almost uniformly exempt, while their calves suffer severely with all the characteristic symptoms of the malady, is likewise considered certain; and all the people quite as firmly believe, that calves never have the disease, except when their dams are suffered to wander into those spots. The belief as a fact, is equally general, that if the calf die of the disease, and the cow is suffered, as happens invariably, to go dry, she herself, often falls a victim to the Trembles. Many persons are satisfied, that they have known the disease produced in men, by eating the flesh of cattle, which had been brought to the slaughter, directly from places which abound in the remote cause; and all are deeply convinced, that dogs and hogs which eat of the carcases of animals dead of Trembles, are liable to contract from that cause, the same disease.

Having stated these points of popular belief, we shall bring forward a number of facts bearing upon them; throwing those which relate to the pernicious influence of the milk on man and the calf, into one group; and placing in an another, those which relate to the effects of the flesh upon man, the dog, the hog, and the buzzard.

From Dr. McGarraugh, we learned the following facts. Henry Boughn, three miles west of Washington, where the Trembles prevails, persuaded his neighbors to keep up their milch cows: all who did so, escaped Sick-Stomach, those who did not, suffered. One of his neighbors, John Coil, kept up his milch cows for five successive years, and his family escaped the disease, he then permitted them to run out, and his daughter and himself suffered attacks of the malady. Afterwards he kept them in pastures, and had no more of it. William Thompson, of the same neighborhood, had Sick-Stomach in his family, for five or six years, during which he persisted in suffering his cows to run at large. At length he fenced off a part of his meadow, and put two milch cows into it, keeping them there for two years, through both of which the family were free from the disease; he then suffered them again to run at large, when two of his daughters were taken down, one of whom died.

The following fact, if not so much to the point, is worthy of record. John smith and his wife, near Martinsburg, Fayette county, in the summer of 1838, experienced attacks of the disease, from which they recovered; but between the 1st of July and the 18th of August, they lost six children, with what Dr. McGarraugh, Dr. Henton and Dr. Dunlap, pronounced the same malady. The youngest child, three years old, was the only member of the family that escaped. We conversed with the parents, and the narrative they gave us of their symptoms and feelings, warrants the conclusion of those gentlemen. Their milch cows, four in number, with their other cattle and the calves, all continued well, however, except a yearling, which had an attack of Trembles, just before the illness of the family. The whole ran at large, and went every morning, into a deeply shaded oak plateau, in which we spent several hours, and found numerous elm and maple slashes. It was, indeed, part of a tract, that was noted for causing the Trembles. Anxious to ascertain the existence of some less doubtful cause for this dreadful mortality, we made many inquiries of the survivors, but could fix upon none. All the family had eaten the milk and butter of these cattle.

Judge Carothers who resides about two miles from Smith, in the following year, had three of his children attacked, two of whom died. One was taken on the 8th, another on the 9th, and another on the 29th of September, He kept his milch cows in meadows, and had not before had the disease in his family; but as the grass of that autumn had been withered by the drought, his cattle were suffered to go into a piece of enclosed woodland, which we found on examination to present the characteristic surface. One of his unweaned calves, had a well marked attack of trembles, but recovered. A few days before his children were seized, a young cow, not giving milk, was taken and died. All his family had eaten milk up to the time of the attack. They did not observe any change in its quality.

Judge Jameson, already mentioned, testified, that he never knew a milch cow to have the Trembles; but that he has repeatedly seen their calves affected and die, when the cows ran in the woods which generated the disease. Long as he has lived in the midst of it, not one of his family has experienced an attack. He has always kept his milch cows on meadows. He has seen more than fifty cases of Sick-Stomach, every one of which cccurred in persons who had eaten the milk or its preparations, or the flesh of cattle that had run in woods, where the cause of Trembles existed.

Mr. Yeoman, of Washington, gave us the following statement of his own case, which was confirmed by his family, several of his neighbors, and his physician, Dr. Henton. In 1831, his only milch cow ran in the woods, which around Washington are well known to cause Trembles. She as usual, with cows that are milked, continued well. On the first of June, as the season for Sick-Stomach was approaching, the different members of his family, apprehensive of the disease, left off the use of milk; but he was incredulous, and took it in greater quantities than before. In the course of that month, he became costive, felt torpid and sluggish, was a little debilitated and occasionally experienced slight nausea and giddiness in the morning. About the 5th of July, after a fatiguing walk, all the characteristic symptoms of Sick-

Stomach were developed, and it was five days before his costiveness was overcome. This part of his case we have already stated, in the history of the disease. The calf of this cow, became affected about the same time, but lived. A pig and a puppy were fed on the milk. The former died with the Trembles, and the latter when greatly reduced, with the same disease, was killed to relieve it of its misery. All the members of his family remained well.

Joseph Bloomer, Esq., living two miles out of Washington, from having travelled as sheriff of Fayette county for eight years, is intimately acquainted with the Trembles. He declared to us, that much as he had seen of it, he had never known it to occur in a milch cow.

Dr. Henton, of Washington, has never known a milch cow to have the Trembles. He has seen more than two hundred cases of the Sick-Stomach, all of which he thinks occurred in persons who had used the milk or meat of cattle, that ran in woods which produced the Trembles. Eight years ago, in the month of October, he had fifteen patients, in three families. All their cattle had run at large.

Dr. Winans declared, that milch cows are proverbially exempt from the Trembles; but their calves are often affected, when their mothers run where that disease is generated. He once saw two calves laboring under it, and as an experiment, took one from its mother and gave it other food—it got well: the other was allowed to suck, and died.

Josiah Ballard resides on the oak plateau, already described as lying east of Jamestown. When he first settled there, thirteen years ago, he had some sheep which he kept in a small tame pasture. One of the ewes, which had a lamb, jumped into the woods, for three or four successive days in the morning, and returned to her lamb in the evening. In four days from the time she first broke out, it was seized with the Trembles, exhibiting the same symptoms as the calf. He has seen other lambs with it since, some of which died. The symptoms were alike in all.

Mr. Ballard, related the following fact of a neighbor, who lived on the plateau a mile east of him. Allen Rakestraw had one milch cow, which was pastured in an enclosure, embracing with a little tame grass, a portion of unsubdued wood land. Three of his family took the disease and all died. They had used the milk.

Dr. Toland has seen colts with the Trembles, before they began to eat; and calves when they were confined in a pen. He has never known a calf to have it, when the cow was on cultivated grass. After a most ample experience, he is convinced, that the Sick-Stomach is always derived from the milk or flesh of the cow. This was not his first opinion.

We have already mentioned the extraordinary prevalence of the Trembles, on the plateau, a few miles west of London, where the elm slashes are of corresponding number. It now remains to say, that the Sick-Stomach has attacked and destroyed great numbers of the people. Robert Turner who has lived twenty-one years in that neighborhood, gave us, assisted in the recollection by Dr. Toland, the names of ten families, living within a mile and a half of his house, in which thirty-five cases of the disease had occurred, nineteen of which proved fatal. Their milch cows all ran in the dense woods of the plateau.

Dr. Houston, formerly, did not believe in the animal origin of the Sick-Stomach, but multiplied observations have made him a convert to that opinion. He mentioned to us his own case as follows:

During his attendance on a family in which the disease had occurred, he was induced, on two successive days, to drink freely of the same kind of milk they had been using. He became costive, and in a few days was down with all the characteristic symptoms of the malady; which proved so violent, that he took two hundred grains of calomel, before it was subdued. The cows supplying this milk, had run in the woods, and some of their calves had the Trembles.

From the experience of many years, in a region which presented him every summer and autumn, with many cases of the disease, Dr. Bradbury is convinced, that the Trembles in sucking animals, and the Sick-Stomach in man, originate in the use of milk. A patient of his, Douglass, was of a different opinion, and, with his family, continued to use the milk of his cows (which ran at large,) after their calves had been seized with the Trembles: almost every member of his family was taken down with the sickness. Another patient, Harper, made it a practice to keep his milch cows on grass, and his family remained exempt. Once, they escaped from their pasture, and after two days were found in an elm and maple slash. His family ate their milk, after they were brought home, and three or four of its members were attacked with the disease. An acquaintance of his, Baker, had a pack of five or six hounds, among which was a bitch with whelps. He, and several of his calves, while using the milk of cows which ran at large in a woodland tract, were seized with the disease; he recovered, but two or three of them died; the hounds ate of their carcases and all were seized with the Trembles, except the bitch—she escaped, but her whelps were all destroyed.

It would exhaust the patience of the Convention, to give all the facts we have collected on this subject. What are the legitimate conclusions to be deduced from those we have stated? They *seem* to be the following:

1. It is *certain* that the sucking calf contracts the Trembles, when the cow frequents the places which produce that disease, she herself, mean while, remaining exempt; and the same thing is true of the colt, the lamb and the puppy, when its mother has eaten of the carcase of an animal dead from the Trembles.

2. It is *highly probable*, that the Sick-Stomach is produced by the same cause.

It is not without the greatest misgivings, that we feel constrained to make these deductions. They are certainly not made, from all the facts that might be collected, by further observation; and many others could, by a course of experiments, be brought forth; and, therefore, they are liable to be overturned. We have stated the first as certain, because the amount of positive testimony (of which we have given but a fraction) is, indeed, very great, and stands unopposed by a single authentic observation, that has come to our knowledge. We have given the second as a high probability, but if the first be true, does it not come near establishing the second? If the milk of the cow, whether she herself be ill or not, can excite a specific disease in her calf, may not the same milk raise a specific disease in man? This question, we apprehend, must be answered in the affirmative; and if so, the probability that the Sick-Stomach is of the same origin with the Trembles in the calf, is greatly heightened; and the propriety of naming the disease Milk-Sickness rendered apparent.

Let us now inquire into the alleged effects of eating the flesh of animals, which have died of Trembles, or been slaughtered while, or soon after, running in woodlands which give birth to that disease; and first—

### *Of Carnivorous Animals.*

Throughout the District, the conviction is deep and unanimous, that dogs which eat the flesh of animals dead from Trembles, are in danger of the same disease; in fact, that they never contract it in any other way. As a consequence of this opinion, it is common to bury or burn up the carcases of cattle, horses and sheep, as soon as they die, and thus the opportunities for acquiring new facts on this point, have become exceedingly rare. Entertaining strong suspicions of the correctness of this opinion, we were led to make extensive and scrutinizing inquiry, the result of which is, that if we believe any thing touching the whole matter, we must believe this. Without making special citations of fact, we may give the names of a few witnesses:—Drs. McGarraugh, Bradbury, Baskerville, Toland, and Winans; the Rev. Jesse Rowe; Judges, Harold, Carothers, and Jameson; and Messrs. Bloomer, Draper, Ballard, Pierce, Boyer, Janes, and Horney. All these gentlemen reside in various parts of the District, are intelligent and respectable, and testify of what had fallen under their own observation. They and many others, also affirmed, that they had never known a dog to have the Trembles, unless some animal had died of that disease in the neighborhood.

Considering this fact as established, we may proceed to say, that many of these witnesses and other persons in the District, state, that they have repeatedly known hogs to contract the disease from the same origin.

As bearing on this point, we shall give the following statement from Mrs. Jeremiah Warder, of Springfield, Clark county, a lady whose philosophical accuracy, is equalled, only by the benevolent interest she has, for several years, displayed in these inquiries.

Early in the month of September last, a calf owned by one of Mr. Warden's tenants, escaped from its enclosure and passed a night in a spot known before to have generated the Trembles. Reclaimed, and placed in a field, it died the following night, but the symptoms which attended its illness are not given. On the succeeding day, the hogs of the farm, found the carcase and soon devoured all except the bones. A few other hogs, the property of a neighbor, were in the gang. The very next morning, when those belonging to the farm were to be fed, it was found that many of them were sick, some dying and others dead. The former were observed to have swollen throats, were very feeble, would stagger and fall when driven about, lie grunting for a while and then rise. Nearly the whole, amounting to more than forty, died. Those from the neighboring farm, which partook of the carcase, also died.

It must be admitted that we have not sufficient evidence that these animals had the Trembles, but the incident shows that hogs *may* be destroyed, by eating the flesh of a dead calf, a fact which goes to the support of the proposition now under discussion.

The testimony in regard to buzzards is limited and unsatisfactory. We may state, however, that several persons, some of whom have just been mentioned, have occasionally met with one of these birds, which after feasting on the carcase of an animal that had died of Trembles, was unable to fly, and appeared to have great muscular infirmity.

### *Of Man.*

If dogs and hogs are injured by feeding on the carcases of animals, which die of Trembles, and calves and other young animals, which suck are made sick, when their dams are exposed to the remote cause of Trembles, we can see no reason, *apriori*, why the flesh of animals thus exposed, may not injure those who may happen to eat it. True, they do not partake of cattle which died from the disease; but if the flesh of *such* be poisonous, it might have been so, for some time before death. Analogy, moreover, supplies us with an argument. If the milk of the cow can cause the Trembles and Sick-Stomach, she herself not manifesting the external symptoms of the disease, it is not, perhaps, unwarrantable to believe, that the flesh of the steer, slaughtered from the infected woods, may originate the disease in those who eat it. On this point we must recollect one, and perhaps the greatest peculiarity of this affection—the influence of exercise, in suddenly bringing on those symptoms, by which it is recognized. Muscular exertion, however, could not give rise to this effect, if the blood and organic system were in a sound and natural condition; bnt in what their departure from such a state consists, it would be vain to conjecture. It may, perhaps, be similar to that of an individual, who has received the impress of the cause of yellow or bilious fever, and in whom the disease is awakened, even after he has withdrawn from the infected spot, by some slight irregularity. Should it be argued, in the case of Trembles, that there is no latent morbid condition, because the animal retains its appetite, we may reply, that in all stages of the disease, up to dissolution, it continues to eat. It may, however, be said, that if the flesh of animals thus predisposed to the malady, is unhealthy, many persons ought to be injured, as cattle are often taken from the thick woods, to be slaughtered for the table. To which we answer, that the danger, and the means of detection, are perfectly understood in the District. Judge Jameson and others informed us, that they never kill a beef from the woods, without first chasing it; that many of his cattle had died of the Trembles on their way to the Cincinnati market; and we learned from other sources, that it is a common practice for purchasers to employ this test. With this precaution, it is not likely, if the flesh of cattle exposed to the remote cause, but not down with the disease, be unhealthy, that many persons would be injured by it.

Let not our speculations on this branch of the subject be confounded with the facts of our Memoir. We are not aiming to prove, by *argument*, that persons *are* injured by eating the flesh of those animals; but merely to show on principle, its *possibility*, as a justification for stating a few alleged facts, which we cannot omit, without neglecting to notice one of the most current opinions of the people of the District.

The following, narrated to us by Dr. McGarraugh, the attending physician, was afterwards confirmed by Judge Jameson, at whose house two of the patients died.

In Washington, Fayette county, many years ago, a butcher, by the name of Grady, went eight or ten miles into the country south-west of the town, where the Trembles have prevailed from the first settlement of the county, and purchased two cattle for beef. In driving them to town, one took the Trembles and was left by the way; the other was driven on, but was so stiff the next morning, it could scarcely walk. In this condition, it was slaughtered and sold to consumers. Six or seven of them, before night, and three several days afterwards, were seized with the Sick-Stomach, of whom five died. The whole of these were Dr. McG.'s patients, and he heard of several others, who were attacked on the day of the purchase, but whom he did not see.

In the neighborhood of Jamestown, two intemperate men, by the name of Reagan, killed a calf affected with the Trembles, and ate of it; the wife and daughter of one of them, did the same. The whole were seized with Sick-Stomach, of whom the men died, but the women recovered. This narrative we received from Dr. Winans; who with many others assured us, that intemperate men, are not only *most* liable to this disease, but more apt to die from it than others.

Mr. Hammond, near South Charleston, killed a beef from a wood known to generate the disease. Three out of his family ate of the meat, and had the Sick-Stomach. Two other families used it, and several of their members experienced attacks. This fact was given us by Dr. Houston.

Other facts of a similar kind, but vaguely stated, we shall not relate; but proceed, in the conclusion of this branch of our subject, to mention two or three, which if true, would show that the carcases of animals, dying of the Trembles, may seriously affect those who handle them.

Some time ago, Enoch Wilkins, a poor man, who lived four miles from South Charleston, on a spot greatly affected by Trembles and Sick-Stomach, lost a fat steer by the former disease. He skinned the animal, and he and his wife and daughter, rendered out the tallow, some of which was made into candles. Soon afterwards they were all taken down with the disease, but recovered. The candles, in burning, emitted an offensive smell. The family declared, that they had not eaten milk, butter nor beef, for several months before. We derived this statement from Judge Harold and Jonathan Peirce.

Many years ago, Lewis Skilling, already mentioned, as living four miles west of South Charleston, lost about the same time, twenty-one head of cattle, with the Trembles. He and his wife skinned them, and rendered some of the tallow. The candles made from it, in burning, gave out such an offensive smell, that they were thrown away. Both of these persons were taken down soon afterwards, and brought to the brink of the grave. They narrated to us their symptoms, all of which were strictly characteristic of Sick-Stomach, in its most malignant degree. The children in this family did not suffer from the disease; nor were there any other attacks among them. Both before and afterwards, his milch cows were kept in meadows. No part of this story could have been prepared for our use, as it was told to us and written down, at a casual meeting on the high road. We assured ourselves, moreover, by inquiry in South Charleston, that the parties were of a highly credible character.

Such statements must necessarily remind the members of the Convention, of the dreadful diseases, which are some time awakened in the practical anatomist, by the slightest puncture with his knife; although putrefaction may not have commenced. In such cases the poison is probably generated during the disease, and not after death.

Two pathological opinions prevail in the District, on the manner in which the milk and flesh of cattle and other animals become poisonous. One, and the most current, is, that a poison swallowed by the animal, is absorbed, and secreted with the milk, or deposited in the flesh. According to this view, the same agent, not decomposed by passing through its system, which causes disease in the animal, likewise occasions it in man and the animals, which are subjected to the use of milk and meat thus impregnated. The other speculation is, that the original poison excites in the animal taking it, an unhealthy secretory action, by which a virus of a peculiar kind is generated. Facts for a satisfactory decision on these hypotheses, do not exist, and we present them, merely as points for future investigation.

### OF THE SPECIAL CAUSE OF THE TREMBLES.

Assuming it to be ascertained, that the special cause of Trembles, in the District, and we travel not beyond its limits, is something peculiar to certain humid spots, provincially called slashes, which are interspersed, beneath the dense oak forests, over the plateaus or table lands of the District, we shall proceed to inquire what does, or may be admitted to exist in them, which can cause such a serious malady.

The possible causes may be thrown into three divisions—those which are of mineral or geological origin—those which result from the decomposition of organic matter—and those which belong to the living vegetable kingdom. The two first may be dissolved in water and drunk, or exhaled as gases, to enter the lungs, or act on the surface of the body—the last may exist in the substance of the plants, and be eaten, or pass off from them as a gaseous exhalation to exert itself upon the surfaces with which it may come in contact.

### I.—*Mineral Poisons.*

These spots repose on a secondary limestone, and can derive from it no poisonous impregnation, not imparted by the same rock elsewhere. It is, moreover, the underlying rock of considerable tracts within the District, where it appears at the surface, while in the table-land it is buried up; and still the former remain exempt, while the latter are liable. It is worthy of remark, however, that blende (or sulphuret of zinc) has been found in one place, as an imbedded mineral. Now the sulphate of zinc is an active substance which readily excites vomiting, and, from the analogy between zinc and lead, we might conjecture, that the latter may exert on the muscular system, effects of a peculiar kind, such effects, for example, as make a prominent part of the symptoms in the Trembles. It is not necessary, however, to dwell on this speculation, as the transformation of these spots into cultivated land, puts an end to the disease, although many of them may continue to be ponds, and afford water for stock.

The strata of diluvial clay and sand, which make the beds of these spots, have not been analyzed, and may, it might be affirmed, contain poisonous substances, soluble in water. The same clay, however, constitutes the sub-stratum of parts of the District, where the disease never occurs; wells, the water of which is salubrious, are dug in it; finally, its influence would remain unimpaired, after the forest was destroyed.

Mineral exhalations of no kind can be admitted, as the cause of this disease, as they would occur equally, in the intervening barrens and prairies, and continue, moreover, after the destruction of the forest, while the disease does not.

We may, then, safely deny a mineral origin to this malady; although the soil has not been analyzed.

II.—*Gases developed by the decomposition of Organic Matter.*

The soil in these spots, is manifestly composed of that washed into them from the surrounding higher parts of the plateau in former times, and of the the remains of many generations of plants, and small aquatic animals, embracing a minute bivalve shell-fish belonging to the genus *Unio*, which have died and been decomposed by putrefaction. As this fermentation is constantly going on, except in cold weather, there must be a continued exhalation of gases or miasms, and to this cause many persons in the District ascribe the Trembles. The principal facts and suggestions in support of the miasmatic hypothesis are—

1. The season of the year when it prevails, being the same with autumnal fever, which the argument assumes to be produced by some kind of malaria.

2. The fact, affirmed by Dr. Baskerville, a zealous advocate of this theory, and supported by Robert Turner, Enoch King and several other respectable farmers, who cite their experience,—that if cattle be kept in pastures and yards at night, and not suffered to lie in the woods, they neither have the Trembles, nor by their milk excite the Sick-Stomach. Whether all cattle and other animals, if thus treated would escape, cannot of course be known. That particular facts of the kind, have been correctly reported, we do not feel at liberty to doubt. It is, certainly in harmony with the existing state of our knowledge, that lying through the night on damp ground in a deep forest, should favor the action of malaria.

3. The places which originate the disease, are precisely such, as we should expect to generate malaria.

4. When they are converted into tilled land or meadow, the disease ceases.

5. The soil of those places, bears a striking resemblance to that of the prairies, which are natural meadows, and which might perhaps be infested with Trembles, but for some correcting influence of the natural grasses with which they abound.

These facts and suggestions, seem at first view to give some plausibility to the miasmatic theory of Trembles; but formidable objections lie against it. The greater liability of cattle that remain in the woods at night, than of those which are kept in open fields, even if it were established as a general fact, which it is not, would not prove that the disease depended on malaria, for that kind of exposure might be only an exciting cause; nor would it explain the exemption of the milch cow. The margins of streams and various other localities within the District, are infected with the diseases which are ascribed to malaria, without generating the Trembles. Finally, the plants which undergo decomposition in those receptacles, are of the same kinds which are decaying in places, where the Trembles do not prevail; though, out of the same materials, placed under similar circumstances, the same gases ought to be formed. We are, therefore, in the present state of our knowledge, obliged to regard this hypothesis as unsatisfactory.

A modification of the miasmatic theory is that, which supposes the poison developed in the decay of the organic matter, to be absorbed by the water, and drunk by the animals; but this relates only to the mode of its introduction into their systems; and after being liable to the objections jnst stated, is still further defective, in having no analogy to support it.

### III.—*Of Vegetable Poisons.*

Rejecting the articles embraced in our first and second divisions, we find ourselves thrown upon the third, and here we are in association with a great majority of the physicians and people of the District. But although they concur in the opinion of a vegetable origin, they differ widely as to the particular plant.

At first view, all the circumstances would seem to be in favor of this division, as furnishing the special cause. The disease begins in herbivorous animals; they feed among plants, many of which are virulent; it is a well known general fact, that cattle sometimes kill themselves by eating noxious plants; many pre-existing facts go to show, that the flesh and secreted fluids of herbivorous animals are often modified by their food, and that substances of unusual color or odor, not unfrequently manifest their presence, or that of some of their elements, in the solids of the body and in the fluids and gases excreted from the kidneys, the skin and the lungs. There is then much plausibility in the opinion, that the Trembles are occasioned by the eating of a plant, while there is nothing physiologically *absurd*, in the hypotheses of of a contamination of the flesh and milk of the animal. Here then we have a substantial foundation to stand upon, and from whence to extend our inquiries after the particular plant. But before we commence, it is proper to refer to an opinion, held by some persons in the District, that the disease may be the offspring of several. This we consider untenable, inasmuch as different poisonous plants vary from each other in their mode of action and effects, and of course, the symptoms vary; but the symptoms of Trembles are remarkably uniform—as much the same, indeed, as those produced by opium, tartar emetic, arsenic or the preparations of lead.

For a plant to produce the Trembles, it must be one that is noxious; that is not very minute, for the cow, when placed in the midst of abundance, is disposed to browse on the larger; it must be acceptable to the taste of cattle, horses and sheep, not to extend the catalogue, or else it will not be eaten by all which contract the disease; it must be in leaf in autumn as well as summer; it must grow abundantly in and around the spots which generate the disease, and sparsely or not at all in the surrounding plateaus, or in other localities of the District; lastly it must be liable to destruction under simple deadening of the timber, harrowing of the ground, and scattering over it the seeds of any of the grasses.

Having laid down these tests, let us proceed to apply them to the different plants which have been charged with the mischief; or which, if not charged, may, from their known activity, deserve to be scrutinized.

From the beginning of our exploration, we had understood, that the spots which the people call slashes, were supposed to be those which generated the disease, and of course we directed a special attention towards them.

To the results of our inspection, already stated, we may here add, that we could not find in one of those spots, a plant either shrubby, climbing or herbaceous, that was new, or that does not grow in other places. This fact of course limits our inquiry, to the influence of known active vegetables, any one of which *might* be the cause of the malady; but was not likely to be, unless it grew there in greater quantities than in other parts of the District. Among the known active herbaceous plants, which we had presumed might, perhaps, exist there, in unwonted luxuriance, were some species of the genera, lobelia, ranunculus, caltha, gillenia, euphorbia, asclepias, and some of the umbelliferæ; but a careful inspection soon convinced us, that with very few exceptions, the members of these genera, were not more abundant and luxuriant in and about those, than other places in which the Trembles do not prevail. Several of them, indeed, as the asclepias decumbens, the gillenia stipulacea and lobelia inflata, all active plants, were rarer than common in those localities; we were compelled, therefore, to abandon the whole, and turn our inquiry upon those which the people of the District have charged with producing the disease, and,

1.—*Of the Eupatorium Ageratoides.* Early in November, 1838, Mr. John Rowe, a farmer of Fayette county, eight miles south of Washington, announced in one of the newspapers of the town, that on the 1st day of October, he had discovered the herb which causes the Trembles in cattle. The same paper contained a brief account of some experiments made by Mr. Rowe, and a notice that others were in progress. About the time of this publication, we received from Mr. Daniel McClean, of Washington, a letter of the same purport, and a box of the plant. On opening it, we at once recognized a familiar species of eupatorium; but to be assured on this point, we gave specimens to Mr. T. G. Lee and Dr. J. A. Warder, of Cincinnati, both cultivators of Botany, who pronounced to be the *e. ageratoides* of Linnæus—*e. urticafolium* of Michaux; one of the common and widely disseminated autumnal plants of the United States. In the following autumn Mr. Rowe was invited by several graziers, of Madison county, to visit them and make experiments upon such of their cattle as he might select; which he did, with such results, as encouraged him, in the following winter, to visit Columbus, and endeavor to bring the subject before the Legislature of Ohio.

On planning the visit to the District, which has given rise to this Memoir, we of course determined to see Mr. Rowe, as early as practicable, and accordingly spent the second day of our sojourn with him—becoming his pupil, and listening attentively to all his statements. He had no written or printed account of his experiments and observations in the preceding autumn, but from his recollections, and from subsequent conversation with Mr. James Porter, one of the farmers who had invited him into Madison, and Dr. Baskerville, who had made a *post mortem* examination of one of the animals sacrificed in his experiments, we collected the following facts: A fluid extract of the eupatorium, given in gill doses, at short intervals, killed a pig in twelve hours. The lining membrane of its stomach bore evident marks of inflammation and mortification. A calf at first refused to eat the herb, but no other food being allowed, at length, it ate plentifully. In about two weeks, it gave evident signs of being affected, and was pronounced by good judges to have the Trembles. Two other cattle, a steer and a calf, ate the eupatorium reluctantly, and were allowed a quantity of oats at the same time. In about a week, they were pronounced, by the farmers who saw them to have the Ttrembles. They died, and the calf was examined by Dr. Baskerville, who found small dark colored spots in the digestive mucous membrane, which, however, was in general white. He thought it softened. The contents of the intestines were dry and scybalous.

We are not aware of the existence of any other evidence, in support of Mr. Rowe's discovery. On our suggesting to him, that his plant grew extensively over the United States, he declared that he knew of two others, which so nearly resembled as to be mistaken for it. To become acquainted with them, we proposed a visit to the woods in which they grew. Those which he designated, four or five in number, belonged without exception to the other genera, and of course bore but a faint resemblance to the eupatorium.

It must be admitted, that the plant on which Mr. Rowe experimented, possesses some active properties, as four animals under its use, died with what were pronounced to be symptoms of Trembles. Still, the mode of conducting the experiments, differed too widely from that in which the animal is likely to eat the poisonous plant, in the woods; and the decision that the animals killed by it, *had* the Trembles, is far from conclusive or binding. We know from much conversation with Mr. Rowe himself, and the farmers generally of the District, that their diagnosis is taken entirely from the debility, stiffness or unsteadiness of the muscular system. Now if a quadruped be ill from any cause, as, for example, an inflammation of the stomach, its muscular function becomes impaired, and when it is driven about, it falters and is liable to fall, as a *man* would if compelled to run or walk under the same disease. A professional scrutiny only can be relied on in such cases. The testimony adduced by Mr. Rowe is, therefore, defective and inconclusive, even if nothing could be found to oppose it; but there are several facts which directly invalidate it.

1. The plant grows in every part of the District, except the prairies, as well as in all the fertile woodlands of the Western states. In the District it is abundant and very luxuriant in the rich, broken lands where the forest has been thinned out, so as to let in the sun, but the Trembles do not occur there. In the barrens, where the soil is not too poor, it is equally common. In both tracts, however, it makes but little show in autumn, the time of its flowering, unless the grounds are enclosed, for the cattle eat it, almost as fast as it grows. As to the spots which generate the the disease, so far from presenting it in any unusual quantity, they actually produce less, for it does not flourish well in damp and shaded situations.

2. Mrs. Boughn, near Wasington, had an open woodland pasture, which from not being eaten down, through the summer, became perfectly white in early autumn, with the flowers of this plant, which had attained the elevation of three or four feet. In this condition, she turned in her stock of cattle and horses, which eat up, as we might say, the whole crop, but they all remained healthy.

Mr. Blue, near Jeffersonville, took us into a pasture field of the same kind, which the year before was in the same state, and was then rented to one of his neighbors, who drove his stock into it. They remained there for some weeks, feeding upon it with the other herbage, but none of them contracted the Trembles.

On the plantation of Richard Douglas, Esq., three miles from Jeffersonville, we saw more than one hundred head of cattle, in a wood-pasture of great extent, where the eupatorium was growing in profusion, in every place which was inaccessible to these animals, while it was reduced in quantity in all others, by being eaten.

Numerous additional facts might be cited, but we consider these sufficient, to prove that we must seek for some other cause for Trembles, than the eupatorium ageratoides.

2.—The *Bignonia Capreolata.* This is a woody and perennial vine, with many branching and tortuous slender limbs, and opposite conjugate or double leaves. It spreads over bushes and small trees, attaching itself by tendrils, which spring from the common foot stalks of the conjugate leaves. It is known among the people, under two names, Creeper and Straight Mercury. It grows in considerable abundance in and about some of the places, which are regarded as the sources of the Trembles.

Mrs. Goodnight, who has resided on the banks of the Rattle Snake Fork of Paint creek, ten miles south of Washington, in Fayette county, more than twenty years ago, lost her cattle and sheep with the Trembles, when she first settled there; and being told that the bignonia was the cause, she had a tangled patch of the vine which existed in their pasture, destroyed, after which the disease did not show itself. The same lady, boiled the shrub in milk, and fed it to a dog, which at the end of a week was said to be attacked with the disease.

Willis Fergus, of the same county, transferred a healthy steer from a field to an enclosure, containing a thicket of this vine, where in a short time he took the disease.

Felty Coil, of the same county, many years since, had two calves seized with the Trembles in winter. By following their tracts it was found that they had been eating largely of this vine, which still retains its green leaves, at that season.

For these facts we are indebted to a manuscript address of Dr. McGarraugh, (President of the Highland Medical Society, and an advocate of the claims of this plant,) obligingly furnished us by its Secretary, Dr. C. C. Sams. It must be admitted, that they merit consideration; and we may add to them that this vine does not grow generally throughout the District. But much additional evidence is required, and some objections lie against its claims.

1. Other plants, known to be virulent, grow in and about the same places, intermingled with the bignonia.

2. It is not known from other observations or experiments, that this plant possesses virulent qualities. The experiment on Mrs. Goodnight's dog is inconclusive, because he might have eaten of the carcase of an animal dead of Trembles. This vine is, moreover, like the bignonia radicans, or common trumpet flower, cultivated in our gardens and shrubberies, and if it possessed deleterious properties, they would in all probability have been disclosed ere this.

3. According to our own observations while the District, this vine does not grow in very great abundance, and is sometimes nearly or quite absent from spots which generate the disease.

4. The observations here presented, were made many years ago, and might have been expected to have been followed by others; but such seems not to have been the case, for in all our journeying through the District, not one was communicated to us.

It cannot, then, on the testimony which now exists, be admitted, that the bignonia is the cause of the Trembles.

3.—Of the *Fungi*. These are a great natural order of plants embracing mushrooms, champignons, toad stools, &c. Dr. Winans has suggested, that some one or more of these plants, may be the cause of Trembles. Many of them are known to be poisonous; especially, when they grow in deep and damp woods. He has observed them to be abundant on the oak plateau, east of Jamestown, already so often referred to, as generating the disease. In the woods where the cattle of John Smith, who lost six children with Sick-Stomach, were known to feed, we saw them in abundance. Four or five species might be recognized, some of which were exceedingly acrid to the taste. This was in September, when the disease generally prevails. We were told by some persons in the District, that they had observed cattle feeding upon them. These circumstances give some probability to the conjecture of Dr. Winans. When we asked him for particular facts, he frankly answered us that he had none. He did not even know that animals subject to the Trembles, ever eat of this tribe of plants, so remote in their nature and appearance from that herbage, which is their natural food. Our enquiries of other persons were answered in the same manner. We must, therefore, regard the hypothesis of Dr. Winans as merely presenting a new subject for investigation.

4.—*Rhus Venenata* of Decandolle. This shrub, the R. vernix of Linnæus and many other botanists, is known by the people under the names of poison sumach, swamp elder, poison ash, swamp dogwood, &c. It is a shrub which grows from eight or ten to fifteen or twenty feet in height, delighting in rich swamps. It is occasionally found, but not abundantly, in the spots which produce the Trembles. Although an active plant, producing in many who handle it, or are even exposed to its exhalations, a severe but temporary disease of the skin, there is we believe, no observation made in the District, that goes to show its agency in the production of the Trembles. Occurring, moreover, around and in the prairies, which are proverbially exempt, and growing to such an altitude as to render its leaves and twigs, to a great degree, inaccessable to sheep and even cattle, no other reason could be assigned for regarding it as the cause of the Trembles, than its reputed virulence, and its presence, to a moderate extent, where the disease prevails.

V.—*Rhus Toxicodendron* of Linnæus. This is the last plant which we propose to examine in connexion with the Trembles. Its botanical history first claims our attention. By Linnæus and the followers of that great man, it was regarded as an humble shrub, of virulent properties, growing in some of the same localities, with what was considered as a distinct species of *rhus*, and called by him *radicans*, from the radicles, by which its ascending stem attaches itself to the loftiest trees. Later botanists have, however, made but one species of the two, and Drs. Torrey and Gray in their great standard work, the Flora of North America, now publishing, have adopted this consolidation; making varieties of the two Linnæan species, and applying to both the specific epithet *toxicodendron*. One, the former of these varieties, presenting a single smooth and unbranching stem, has received the popular name of poison oak—the other, a climbing vine with many branches, is called poison vine and poison ivy. They have no claim, however, to be regarded even as *varieties;* for, as we ascertained, while in the District, they are but different stems from the same root. This was done by detaching the variety *radicans*, or poison vine, from the trunk of the tree, and tearing up its roots, when stems of the variety *toxicodendron*, or poison oak, came up attached to them; being, in fact, but scions, like those which the white flowering locust (*robinia pseudacacia*) is known to send up; and which no botanist would think of erecting into a separate variety from the tree itself. It is true that these separate stems or scions of the *rhus*, are without radicles; but so are the limbs or branches of the main trunk of the ascending vine. These branches, however, when they grow into contact with a solid body, or even happen in crossing each other to touch, immediately send out radicles; and the stems of the scions, whenever they find a solid support, immediately do the same. It seems, indeed, to be a law of the vegetation of this plant, that it sends forth radicles, alike above and below the surface of the ground, when in contact with solid matter, but never to produce them, in the absence of such contact, when they could be of no use to the plant.

When the *R. toxicodendron* grows in dry situations and hard ground, it sends up few or no shoots; and the, so called, *poison oak* disappears; but when it finds itself radicated in a rich, loose, and permanently wet soil, it sends out its horizontal roots far and wide, from which start up numerous shoots, that rise to the height of two or three feet, and present a shrubbery of what is called poison oak.

Now, it is precisely under these circumstances, that we find the *R. toxicodendron,* in the slashes of the oak plateaus, where the Trembles are generated. And the *number* of vines is so great, as to encircle and garnish a majority of all the trees which grow in these fertile spots.

By these statements and explanations, we are prepared to inquire into the validity of the opinion, that this plant is the cause of Trembles. This may be said to be the *popular* opinion of the District. An aged and respectable farmer, three miles South Charleston, whose name we did not record, informed us, that more than thirty years ago, when he first emigrated to Ohio from Kentucky, he followed, in the snow, the track of several horses, to a pond where they went for drink, and found that they had eaten liberally of the tender stems, of what he called the poison oak. They were soon afterwards seized with the Trembles. We mention this fact, chiefly to show the antiquity, in the District, of this opinion. That it has been cherished so long, and by so many, is some evidence of its truth. But we cannot allow that it rests upon positive observations and experiments. We shall proceed to state such of the facts and arguments on both sides of the question, as were collected, or occurred to us while in the District, beginning with those which oppose the opinion.

1. It has been said, that this plant grows in various parts of the District, where the Trembles do not occur. To this we reply, that they present but few slashes, have not much of the climbing vine, and from the condition of the surface, it sends up but few scions. It is not, therefore, within the reach, or is much *less* within the reach, of herbivorous animals, than in those tracts where the Trembles prevail.

2. Many cattle run on the slashes where the scions of the *Rhus* grow abundantly, without contracting the disease. But it does not follow, that all herbivorous animals, which go at large, will eat the *Rhus*. It has, moreover, this peculiarity. Its poison affects only a part of the people who handle it; and the same poison, may only affect a part of the animals that eat it. This objection, however, may be raised against any other plant; or, indeed, any cause whatever, with as much propriety as against the *Rhus*. Of the inhabitants residing in the same region, some in autumn will escape bilious fever, and others be taken down, while all are equally exposed.

3. Dr. McGarraugh states, that a gentleman in Washington, a few years ago, enclosed a large woodland pasture, adjoining the town plat, in which there were several acres overspread with this vine. It was eaten down by his cattle, all of which, however, remained well. To this fact, we may add, that in the latter part of the September last, Mr. Albert Douglass, a student of medicine, at our request, when sojourning on his father's farm, in Fayette county, subjected a steer to the use of this plant, mixed with hay, for ten days, without any injurious effect, although the animal ate it freely. On the former of these facts we may remark, that as all the woodlands about Washington have been charged with producing Trembles, and are, as we know not only from the growth of the *Rhus* upon them, but from personal ohservation, precisely of the kind which generates the disease, the experiment is as valid against every other cause as against the *Rhus*. Of the second, we may say, that before the experiment was commenced, the leaves had been touched by frost, and might have lost their activity; and that the animal might have had a peculiarity of constitution, which rendered it as insusceptible to the action of the poison as was the person who gathered the leaves, to its action on his skin.

4. There is no conclusive evidence of a single case of Trembles, having been produced by the *Rhus;* which militates against the theory, inasmuch as the abundance of the plant, and the long period through which the attention of the people of the District, has been turned upon it, might have been expected to bring out some well authenticated case.

We shall now proceed to consider the affirmative, in doing which, we shall bring this plant to the tests which have been laid down.

1. It exhales a noxious effluvium and appears to contain a poisonous juice.

2. It is of a proper size to be eaten by, while it is accessible to, all the herbivorous animals, which are subject to the disease.

3. Cattle and horses are known to eat it, when not constrained to do so by the want of other food.

4. It is in leaf in summer and autumn when the disease chiefly prevails; and its pith and tender stems, may be eaten in winter.

5. It grows abundantly in and around the spots which appear to produce the disease; and most abundantly where the disease has prevailed most; as on the plateau west of London; while it is scarce in all those portions of the District, from which the disease is absent.

6. By cutting down or deadening the trees to which the *Rhus* attaches itself, and by breaking up the surface of the ground, the whole plant is immediately destroyed, and with this change the disease disappears.

Thus the *Rhus toxicodendron* stands the whole of our proposed tests. Does this, however, prove it to be the cause of Trembles? Certainly not, but it shows, that this plant *may be* the cause, and renders the popular opinion of the District highly probable.

### PREVENTION OF THE TREMBLES AND MILK-SICKNESS.

According to the facts and views of this Memoir, the prevention of Milk-Sickness within the District, (and we shall not extend our conclusions beyond its narrow limits) depends on securing milch cows and beef cattle, from the action of the cause of Trembles. This may be done, either by confining them to cultivated pastures, where they are always safe, or by destroying the cause, when they might run at large with equal impunity. The confinement of milch and beef cattle to cultivated fields, can be neither difficult nor expensive, to any but pioneers of the forest; and if the evil were limited to them, the subject would scarcely deserve further investigation. The Trembles, however, destroy cattle, horses, hogs and sheep, which constitute a large portion of the personal property of the farmers of the District, few of whom are or can be prepared, to pasture the whole of their stock; and hence the necessity, if possible, of extirpating its cause. The people have constantly assumed, that if the cause could be discovered, it could of course be removed. But this might or might not be the case. Suppose it were a mineral impregnation of the water? it could not be corrected; or malaria? its generation could not, in all probability be prevented; or a plant, disseminated among others? it could not be eradicated, leaving them behind. Our inquiries have led us to the last as the most probable conclusion; and we have made some efforts to discover the particular species, but these efforts were instigated more by the desire to gratify popular and scientific curiosity, than under the conviction, that when discovered it could be destroyed by any other means, than those which would, at the same time, destroy its companions of the forest. With these views before us, we must regard the discovery of the kind of *localities*, which give rise to the disease, as the greatest that could be made; and the *only* one which is necessary, to the choice and execution of the requisite measures of prevention.

Now, throughout this Memoir, we have almost adopted the opinion, that the elm and *Rhus* slashes of the oak plateaus, and these alone, are the abode of the special cause of the Trembles; but candor requires us to say, that this has not been conclusively proven; nor is it the opinion of *all* the inhabitants of the District, for we met with several intelligent and observing persons, who believed that the drier and more extensive portions of the plateaus, and they only, generate the special cause.

The final decision of this question cannot be made without additional facts; the acquisition of which cannot be easily

6 *

made, from the great number of slashes, and the consequent difficulty of making seperate experiments upon them, and the other parts of the plateaus. If it should be found, that the cause is in the latter, the means of prevention will be too expensive to be employed in advance of their full settlement; but if it exists in the slashes, and there only, it might be destroyed with great facility, and at a moderate expense, by clearing and cultivation. It is not even necessary to cut down the timber and clear it off, to bring about the desirable security. Deadening it and letting in the sun, answer the purpose, espcially if the spots be sown with the seeds of any of the grasses. The effect of this deadening is to kill the *Rhus;* not merely its ascending stem, which is necessarily cut through in the process of girdling the tree, but also the root, and with it, as a matter of course, the shrubbery of scions called poison oak. Thus, with one day's labor, a single man, might not only destroy all the poison oak in many of these slashes; but set on foot an extensive change in its vegetation, which in a couple of years would be completed without any other labor; though the result would be rendered more certain, by foddering cattle upon them for a winter, or harowing the surface, or mowing down the weeds, and sowing it with grass seed.

In conclusion we may say, that if these spots generate the disease, it could be of no practical utility to know that *a* plant is the special cause, much less to know the *particular* plant, if it has not already been discovered in the *Rhus;* for it could not be destroyed in any other way, than that which has been pointed out—a method which, from much personal observation in the District, we are persuaded is infallible.

